# Effect of Cyclic Loading on Surface Instability of Silicone Rubber under Compression

**DOI:** 10.3390/polym9040148

**Published:** 2017-04-21

**Authors:** Zhonglin Li, Zhiheng Zhou, Ying Li, Shan Tang

**Affiliations:** 1 College of Materials Science and Engineering, Chongqing University, Chongqing 400017, China; zhonglin_li@cqu.edu.cn; 2 Department of Engineering Mechanics, Chongqing University, Chongqing 400017, China; zhihengzhou@cqu.edu.cn; 3 Department of Mechanical Engineering and Institute of Materials Science, University of Connecticut, Storrs, CT 06269, USA; 4 Department of Mechanics, Dalian University of Technology, Dalian 116024, China

**Keywords:** cyclic loading, silicon rubber, creases, surface instability, finite element simulation

## Abstract

This work combines experiments and finite element simulations to study the effect of pre-imposed cyclic loading on surface instability of silicon rubber under compression. We first fabricate cuboid blocks of silicon rubber and pinch them cyclicly a few times. Then, an in-house apparatus is set to apply uniaxial compression on the silicon rubber under exact plane strain conditions. Surprisingly, we find multiple creases on the surface of silicone rubber, significantly different from what have been observed on the samples without the cyclic pinching. To reveal the underlying physics for these experimentally observed multiple creases, we perform detailed nanoindentation experiments to measure the material properties at different locations of the silicon rubber. The modulus is found to be nonuniform and varies along the thickness direction after the cyclic pinching. According to these experimental results, three-layer and multilayer finite element models are built with different materials properties informed by experiments. The three-layer finite element model can excellently explain the nucleation and pattern of multiple surface creases on the surface of compressed silicone rubber, in good agreement with experiments. Counterintuitively, the multilayer model with gradient modulus cannot be used to explain the multiple creases observed in our experiments. According to these simulations, the experimentally observed multiple creases should be attributed to a thin and stiff layer formed on the surface of silicon rubber after the pre-imposed cyclic loading.

## 1. Introduction

Elastic surface instability of silicon rubber has been harnessed to realize the recoverable large deformation of flexible electronics for silicons, carbon nanotubes and graphenes [1,2,3,4]. When a homogeneous silicone block is compressed, its smooth surface suddenly folds into a region of self-contact with a sharp tip when a critical strain is attained, thereby forming creases [5,6,7]. Despite the abundance of crease formations in scientific and engineering applications, the underlying physics principles emerge only recently with the nature of creases being attributed to the broken symmetry of scale and translation [8]. With this regard, creases can be distinguished from the often discussed wrinkles [9,10,11,12,13,14]. When silicone rubber is used in engineering applications, it often experiences cyclic loadings. Thus, a question naturally raises: can cyclic loading influence its surface instability behavior? Under cyclic loading conditions, few studies show that rubbers such as Nitrile Butadiene Rubbers (NBRs) exhibit a stress-softening response [15,16,17]. However, up to now, the effect of cyclic loading on the surface instability of rubber has not been answered. To understand this effect, we need to resort to experiments to study the surface instability after the cyclic loading applied on rubber specimens.

Experimental study on surface instability of silicone rubber under mechanical compression remains difficult and challenging because barreling instabilities usually occur at much lower strains, compared with the critical strain for creasing. To circumvent this issue, researchers have resorted to bend a slab or rod [18,19]. Nevertheless, quantitative characterization on these geometries is complicated, induced by both nonuniform (inhomogeneous) deformation along the thickness direction of specimens and difficulty of measuring spacing and shapes of the surface folds. Cai et al. [20] and Chen et al. [21] have studied the nucleation and growth of creases by compressing a soft elastomer (Polydimethylsiloxane, PDMS) on a stiff substrate (PDMS) and introducing material variation along thickness direction from the edge to center of the thin film, which can introduce creases to nucleate at the edge and channel towards the center. However, when the substrate is introduced, the measured critical strain and pattern can greatly differ from those of homogeneous soft solids. The consideration of the variation of modulus along thickness direction due to the bilayered structure is also numerically and experimental challenging. Such a barrier can be resolved by developing an in-house loading apparatus. In this study, we experimentally demonstrate that the nucleation and progressing of creases on the surface of a cuboid silicone block subjected to mechanical compressive loading under plane strain conditions can be monitored though this in-house loading apparatus.

In particular, with this in-house loading apparatus at hand, we can study the surface instability after the silicone rubber experiences cyclic loadings. Since the silicon rubber is very soft, we pinch the silicone rubber cyclicly a few times (usually 10 times) by a hand. It is followed by putting the specimen into the loading apparatus. When the imposed compressive strain is around 30%, multiple creases are formed on the top surface. It is extremely surprising because the critical strain for nucleating a single crease on a homogeneous soft solid is around 35% [5,22], for wrinkles around 46% given by the classical solution of Biot [23]. The pattern of the surface creasing is also different from a single or double crease, which is typically observed in our experiments for specimens without cyclic loading. Therefore, it is interesting to probe the underlying mechanisms for the formation of multiple creases.

In this work, we combine experiments and finite element simulations to study the effect of pre-imposed cyclic loading on surface instability of silicon rubber under compression. This paper is organized as follows. We first explain our experimental setup with more details for uniaxial compression under exact plane strain conditions. The nanoindentation test is also performed to examine the modulus of silicone rubber at different locations. Experimental results are presented in Section 2. In Section 3, we propose both three-layer and multilayer finite element models to study the compressive behavior of silicone rubbers with pre-imposed cyclic loading. Results and discussion are given in Section 4. Finally, the main results and conclusions are summarized in Section 5.

## 2. Experimental Setup and Results

Our silicone rubbers are prepared with Part A (vinyl silicone, white carbon black and organic platinum catalyst) and Part B (vinyl silicone and white carbon black). Part A and Part B are dispensed in a 1:1 volume ratio (25 mL and 25 mL for part A and part B, respectively). In addition, 1-Ethynyl-1-cyclohexanol (5 mL) is also added to delay the solidification process. Part A, Part B and 1-Ethynyl-1-cyclohexanol are thoroughly mixed for three minutes by manual stirring. The mixed material is put into a vacuum drying oven to eliminate any entrapped air. Then, it is poured into cuboid glass molds with different sizes.

**Figure 1:**
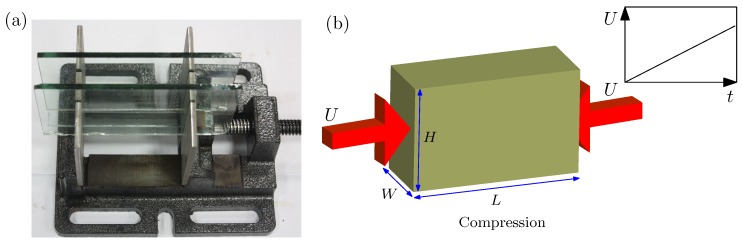
(**a**) in-house loading apparatus to realize the exact plane strain condition under uniaxial compression; (**b**) schematic of the monotonic uniaxial compression. The size of the specimen is denoted by length *L*, height *H* and width *W*.

For the experimental setup, an in-house loading apparatus is designed in which a cuboid specimen of silicone rubber with length *L*, height *H* and width *W* can be placed, as shown in Figure 1. For all the specimens used in present study, L=4 cm, height H=2 cm and width W=2 cm. An example is given in Figure 1b.

**Figure 2:**
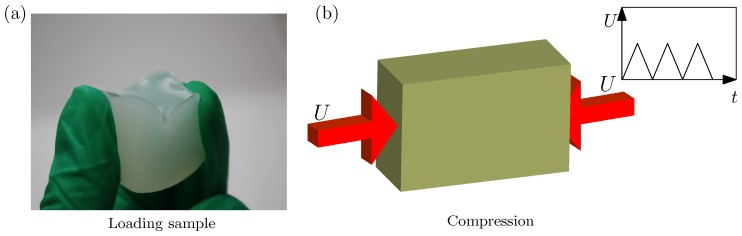
(**a**) cyclic pinching on a cuboid silicone rubber; (**b**) schematic of the cyclic pinching. The loading-unloading is controlled by the imposed displacement.

Before the uniaxial compression, we first pinch the specimen by a hand until creases are formed on the surface (cf. Figure 2a). Note that there is usually a single crease on the surface. Afterwards, the compressive loading is released slowly and the specimen returns to its original configuration. This loading-unloading process is repeated around 10 times, as shown in Figure 2b.

After the cyclic loading, we then place the specimen into the loading apparatus for uniaxial compression. The compressive loading is imposed manually such that the loading rate is extremely low, where the strain is imposed from 0% to 30% over 60 s; the strain rate is approximately 5 × 10^−3^ s^−1^. The application of compressive loading is accomplished by a pair of screw-driven plates placed width-wise of the specimen. The experimental uncertainty in strain is around 0.01 (in comparison, Gent and Cho [18] have reported an uncertainty of 0.07); spiral distance/pitch of the loading rod is measured to be 1.0 mm and the precision of the vernier calliper is 0.01 mm. Two glass plates are placed length-wise of the specimen and are held fixed to impose plane strain conditions exactly. Common washing liquids are applied to the contact surfaces of plates and specimen to reduce friction between them. Washing liquids are effective to reduce the friction, which has been shown in our recent work [22]. The imposed compressive strain can then be easily measured by the length of the specimen during deformation. This method of imposing plane strain conditions differs from what have been done previously. In these experiments [24], a specimen with a large width is usually employed to implement the plane strain condition. However, such a condition is only applicable to a thin slice of the specimen, typically located at the specimen’s longitudinal centerline; overall, plane stress condition still prevail. The current methodology of utilizing glass plates to constrain the specimen ensures that the deformation of the finite-width specimen is under exact plane strain conditions, which is also discussed in our recent work [22].

**Figure 3:**
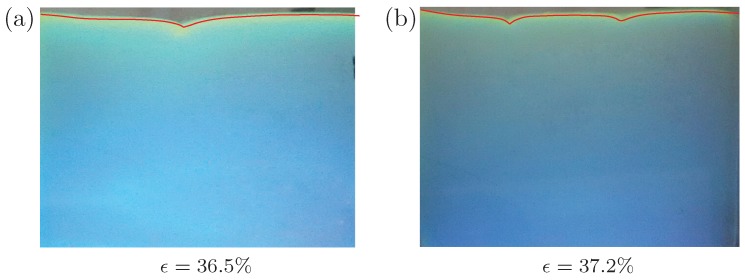
Creasing pattern for a cuboid silicone rubber under the uniaxial compression and plane strain conditions. (**a**) a single crease at engineering compressive strain 36.5%; (**b**) double creases at engineering strain 37.2%. The silicone rubber does not experience cyclic loadings before the uniaxial compression.

For an identical specimen without pre-imposed cyclic loading, the uniaxial compression under plane strain conditions is carried out for comparison. The detailed results are presented in our recent work [22]. Typical results are given in Figure 3 to compare with the specimens after pre-imposed cyclic loading. According to Figure 3, the critical strain ϵ=36.5% is required to nucleate a single crease and 37.2% for double creases (it is proved by numerical simulations that the nucleation strain for double creases is slightly larger than that for a single crease [22]). The same experiments are repeated many times and only two patterns, i.e., single crease or double creases, are observed throughout all of these experiments. The probability for the appearance of a single crease is higher than that of double creases.

**Figure 4:**
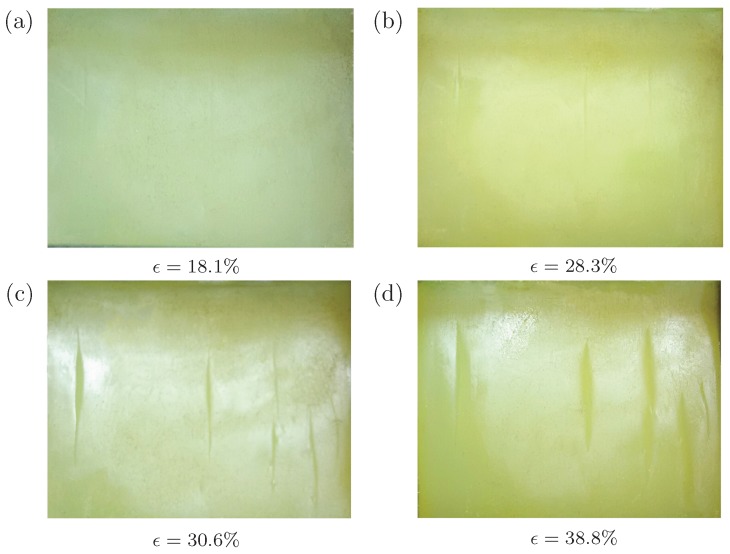
Surface morphology of a cuboid silicone rubber under different levels of compressive strains: (**a**) 18.1%; (**b**) 28.3%; (**c**) 30.6%; (**d**) 38.8%. The specimens experience cyclic loadings before the uniaxial compression.

We then show the pre-imposed cyclic loading can lead to a different creasing pattern. Figure 4 plots the surface morphology of the silicon rubber under different levels of compressive strains, ranging from 18% to 40%. After around 10 cycles of loading-unloading process, very shallow ‘scars’ are left on the surface, as shown in Figure 4a. Laser-scanning confocal microscopy (LSCM) is employed to measure the morphology of the top surface of the specimen quantitatively. However, we still cannot identify the depth of these ‘scars’ by LSCM. It suggests that the depth of these ‘scars’ should be less than 0.01 mm, which is the minimum resolution of LSCM equipment provided by supplier. A similar phenomenon has been observed for PDMS [21]. Scars then grow into shallow creases at a strain around 28% to 30% (see Figure 4b,c). With the increasing compressive strain, these creases become wider and deeper (see Figure 4d). The evident multi-crease patterns can be observed by the naked eyes, significantly different from the patterns given in Figure 3. In addition, comparing with critical strains for single or double creases shown in Figure 3, the strain required to nucleate multiple creases usually should be larger because they encounter higher energy barriers than that of single or double creases. However, against our intuition, the critical strain for nucleation of multiple creases (28–30%) for the specimen with pre-imposed cyclic loading is lower than that without pre-imposed cyclic loading (36.5% and 37.2%).

It should be emphasized that the cyclic pinching cannot be controlled quantitatively because the loading is imposed by a hand. In this paper, we focus on two characteristics induced by cyclic pinching: the appearance of multiple creases and the observed critical strain 28% for the occurrence of multiple creases is smaller than that of single or double creases (the critical strain is around 35%). Several different persons in our group did the experiments on the same material separately. The imposed force during the cyclic pinching may not be the same by different persons, but experimental results show that these two characteristics can still be observed. In this way, the experiments’ results are repeatable and reproducible.

**Figure 5:**
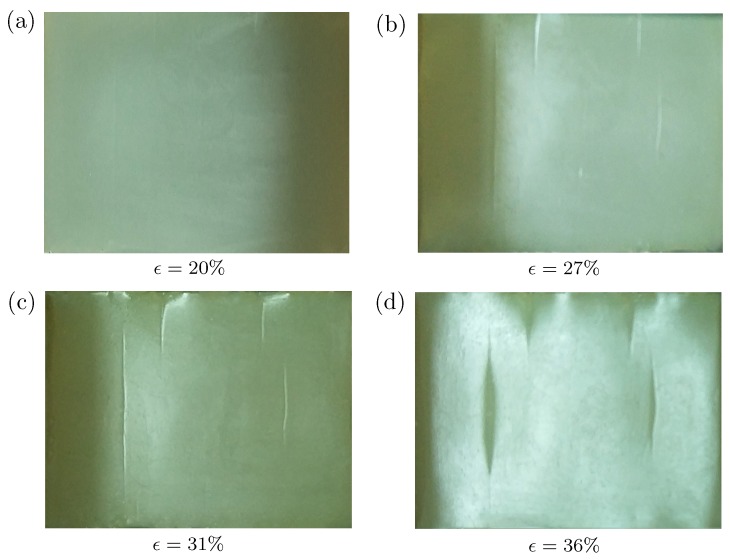
Surface morphology of a cuboid silicone rubber (Ecoflex 00-20 from Smooth-on Inc., Pennsylvania, PA, USA) under different levels of compressive strains: (**a**) 20%; (**b**) 27%; (**c**) 31%; (**d**) 36%. The specimen experiences cyclic pinching before the uniaxial compression.

To further prove that the above two characteristics are general, we also synthesize silicone rubber Ecoflex 00-20 from Smooth-on Inc., Pennsylvania, PA, USA, which is widely used by many other researchers, especially for flexible electronics. The fabrication method for Ecoflex silicone rubber is also the same as aforementioned. We performed cyclic pinching first, followed by uniaxial plane strain compression. The surface morphologies under different levels of the imposed strain are shown in Figure 5. The multiple creases are also nucleated on the surface around the imposed strain level 27–31%. Thus, we conclude that the experimental observations are repeatable because the above two characteristics are observed again. In the following, we focus on the silicone rubber of the first kind and results for Ecoflex silicone are not shown.

**Figure 6:**
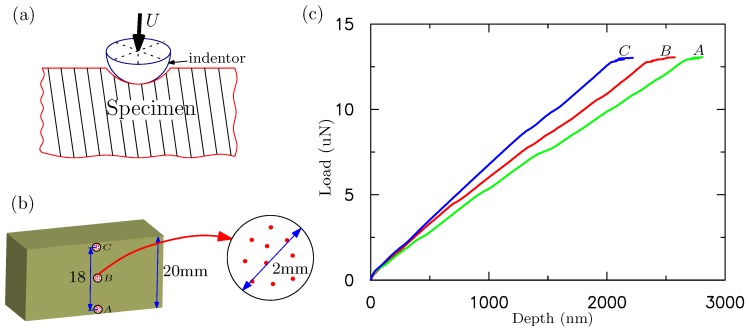
(**a**) schematic of nanoindention test; (**b**) measured load vs. (**c**) indentation depth at the different locations on the cuboid silicone rubber along height direction. Different locations are marked as ‘A’, ‘B’, and ‘C’ on the specimen shown in the insert.

To understand these multiple creases on the surface of silicone rubber with pre-imposed cyclic loading, we perform nanoindentation experiments on these specimens before and after the cyclic pinching. The nanoindentation experiments have been performed by using Hysitron (TI-950) Minneapolis, MN, USA. The tip of the indenter is spherical with a radius of less than 40 nm shown in Figure 6a. To test the homogeneity of the material, nanoindentation tests have been performed at three different locations marked as ‘A’, ‘B’ and ‘C’ on the sample in Figure 6b. The indentations have been carried out at ten random points within a circle (radius 2 mm) around ‘A’, ‘B’ and ‘C’, respectively. The measure forces and depths at ‘A’, ‘B’ and ‘C’ are averaged over the results from these ten points. The force vs. depth curves obtained from nanoindentation experiments are presented in Figure 6c. The indentation experiments for ‘A’, ‘B’ and ‘C’ are also repeated three times. The results are almost the same. The specimens are found to have inhomogeneous mechanical properties because the modulus at location ‘A’ is smaller than that of ‘C’ as shown in Figure 6c. Before the cyclic pinching, the specimen is almost homogeneous as expected. Therefore, we can conclude that the cyclic pinching can change the homogeneous material into inhomogeneous one.

## 3. Finite Element Simulations

### 3.1. Material Models

The homogeneous silicone rubber without experiencing cyclic loading is tested under uniaxial tension to establish its stress–strain curve, so that an appropriate constitutive model can be chosen in the subsequent finite element (FE) simulations. We find that the silicone rubber does not fracture at the tensile strain around 700%, indicating a hyperelastic behavior. In view of this, a hyperelastic constitutive model can be employed to describe the silicone rubber’s elastic behavior in our FE simulations. These simulations are performed by using the commercial FE simulation package ABAQUS (User’s Manual Version 6.13, Providence, RI, USA) [25]. To describe the hyperelastic behavior of silicone rubber, we adopt the compressible neo-Hookean material model with following free energy density:

(1) $$W_{e}=\frac{\mu }{2}\left({\bar{I}}_{1}-3\right)+K{\left(J-1\right)}^{2},$$

where J=detF, $\bar{F}=J^{-1/3}F$ and ${\bar{I}}_{1}=J^{-2/3}I_{1}$. $I_{1}$ is the first principal invariant of the left Cauchy–Green tensor $C=F^{T}F$ with F the deformation gradient and μ and *K* the shear and bulk moduli, respectively. At the small deformation regime, the hyperelastic material can be reduced to a linear elastic material. Consequently, different Poisson’s ratios can be realized by adjusting the ratio between μ and *K*, as Young’s modulus *E* and Poisson’s ratio ν can be related to μ and *K* through the classical relationships E=2μ(1+ν), ν=(3K−2μ)/[2(3K+μ)] [26]. The first Piola–Kirchhoff stress can be obtained by [27]:

(2) $$P_{iJ}=\frac{\partial W_{e}}{\partial F_{iJ}}$$

for compressible hyperelastic solids. To model the incompressible hyperelastic materials, we fix the Poisson’s ratio to be 0.499 (nearly incompressible).

To understand the viscous effect on the formation of multiple creases on the surface of silicone rubber with pre-imposed cyclic loading, we adopt the phenomenological model proposed by Bergerstrom and Boyce [28], which has been implemented into ABAQUS already. The details are given in the ABAQUS theory manual [25]. However, more elaborate viscoelastic models based on the motion of the polymer chain can also be used such as Tang et al. [29] and Li et al. [30,31].

### 3.2. Surface Instability Analysis

**Figure 7:**
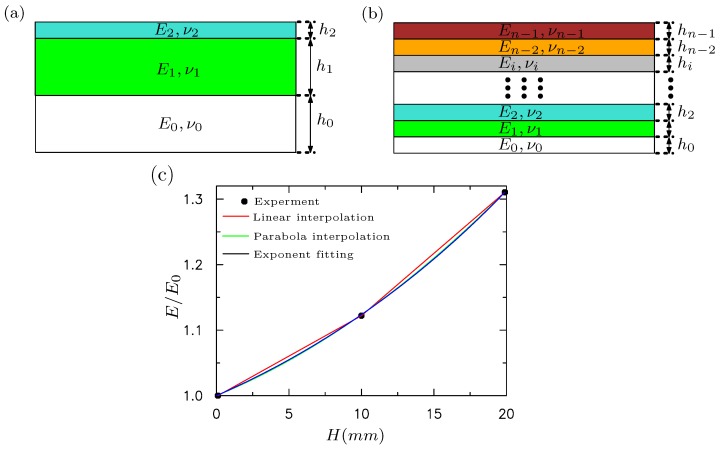
(**a**) schematic for the three-layer finite element model. The height for each layer is $h_{1}$, $h_{2}$, and $h_{3}$ with modulus $E_{0}$, $E_{1}$ and $E_{2}$, respectively; (**b**) schematic for a multi-layer finite element model with n=200 layers. The height of the *i*th layer is $h_{i}$ with modulus $E_{i}$. The height of each layer ($h_{i}$) is equally distributed in the simulations; (**c**) the normalized modulus vs. height for the multilayer model based on linear interpolation, parabola interpolation and exponential fitting. The elastic modulus is normalized by $E_{0}$.

Both three-layer and multilayer FE models are built to study the surface instability of inhomogeneous silicon materials after the cyclic loading. The FE models consider the same dimension of these materials as experiments. These FE models are shown in Figure 7. The plane–strain element CPE4 is used in all of the simulations. The total numbers of elements and nodes for the FE model are 80,000 and 80,601, respectively, following the mesh converge study. For the three-layer model, three different layers with heights $h_{0}$, $h_{1}$ and $h_{2}$ are bonded perfectly together without the interface separation. All three layers behave like a hyperelastic-viscous material with Young’s modulus $E_{0}$, $E_{1}$ and $E_{2}$, corresponding to that measured at locations ‘A’, ‘B’, and ‘C’, respectively, given in Figure 6. For the multilayer model, we assume that the moduli for each layer varies. The modulus of the *i*th layer is denoted by $E_{i}$. Linear interpolation, parabola interpolation and exponential fitting are used to fit the moduli at locations ‘A’, ‘B’, and ‘C’ shown in Figure 6. For simplicity, the modulus is normalized by $E_{0}$. In the following, $E_{0}$ is fixed as 1, and $E_{1}=1.12$ and $E_{2}=1.31$, obtained through the slope of curves shown in Figure 6c. The normalized modulus vs. the height for multilayer model is shown in Figure 7c, based on linear interpolation, parabola interpolation and exponential fitting. For the viscous deformation, the default values of ABAQUS for the parameters involved in the Bergerstrom–Boyce model [28] are employed.

To fully understand the surface instability (creasing), we carry out the FE analysis through a two-step analysis: (1) buckling followed by (2) post-buckling analysis. Buckling analysis for finite sized domains is described in the ABAQUS theory manual and previous works [10,32,33,34,35]. After determining the buckling modes from linear instability analysis, an imperfection in the form of the most critical eigenmode is introduced into the FE mesh. The mesh is perturbed by the corresponding eigenmode and scaled by a factor ω. It has been shown by Ref. [32] that the surface morphology is imperfection-sensitive. However, our results demonstrate that when the imperfection is large enough, the results appear the same for theses simulations. Therefore, the imperfection is set as w=0.003H by choosing ω=0.003.

## 4. Results and Discussion

**Figure 8:**
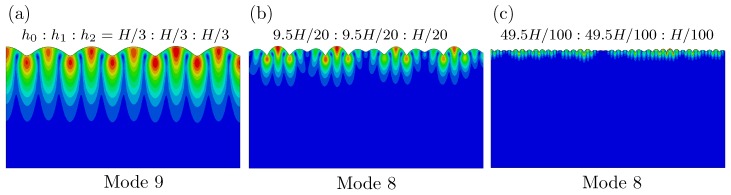
Buckling modes for three-layer models with different heights of each layers. (**a**) case I $h_{0}$:$h_{1}$:$h_{2}=H$/3:*H*/3:*H*/3; (**b**) case II $h_{0}$:$h_{1}$:$h_{2}=9.5H$/20:9.5H/20:H/20; (**c**) case III $h_{0}$:$h_{1}$:$h_{2}=49.5H$/100:49.5H/100:*H*/100.

We first perform buckling analysis on a three-layer model. Figure 8 shows the buckling modes for three-layer models with different heights. They are represented by case I $h_{0}$:$h_{1}$:$h_{2}=H/3$:H/3:H/3, case II $h_{0}$:$h_{1}$:$h_{2}=9.5H/20$:9.5H/20:*H*/20 and case III $h_{0}$:$h_{1}$:$h_{2}=49.5H$/100:49.5H/100:*H*/100 in Figure 8a–c, respectively. Modes 9 and 8 are given for cases I, II and III, respectively. From these results, it is evident that the smaller height of the top layer ($h_{2}$ of case III) leads to more wrinkles on the surface. The other effect of the thinner top layer is that the amplitude of the wrinkles becomes more nonuniform, comparing case III with other cases.

**Figure 9:**
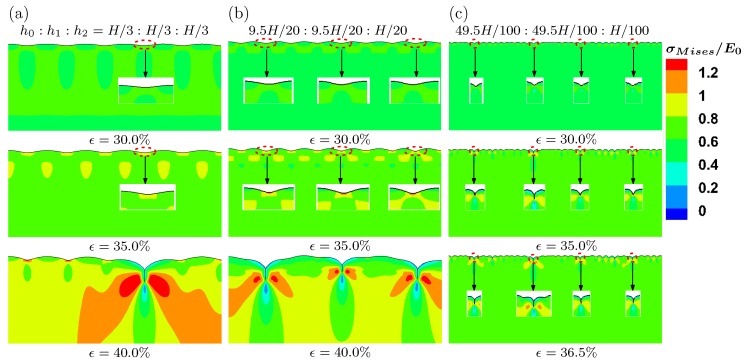
Evolution of surface morphology for a three-layer model at three different levels of compressive strain. (**a**) case I $h_{0}$:$h_{1}$:$h_{2}=H/3$:H/3:H/3; (**b**) case II $h_{0}$:$h_{1}$:$h_{2}=9.5H/20$:9.5H/20:H/20; (**c**) case III $h_{0}$:$h_{1}$:$h_{2}=49.5H/100$:49.5H/100:H/100.

The buckling analysis is a linear analysis procedure. The creasing, a local instability phenomenon, cannot be obtained through such a linear analysis method. Nevertheless, the buckling modes can be used as an input to obtain complex surface morphologies including creasing, which is induced by loss of stability through post-buckling analysis. At first, the viscous effects are excluded by setting a slow strain rate $2.5\times 10^{-5}$ s^−1^. The evolution of surface morphology under different levels of compressive strain is plotted for cases I, II and III in Figure 9. The color represents the von Mises stress distribution. The top surface is perturbed by the buckling modes shown in Figure 8. When the compressive strain increases to 30%, for cases I and II, the surface still remains as the sinusoidal wave, which are clearly shown in the inserts by zooming in the corresponding regions on the surface. For case III with a thinner top surface layer ($h_{2}$), four troughs of waves begin to form cusps at strain 30%, which agrees with experimental measured critical strain for multiple creases. Then, four cusps fold to form multiple creases, a region of self-contact with a sharp tip at strain 35%. The location and number of these creases on the surface in case III seem to be consistent with experiments (see Figure 4). For cases I and II, the full creases are formed around strain 41%, which is much higher than that of case III. The number of these creases is also less than what have been observed in experiments. Therefore, we can conclude that the height of the top layer plays an important role on the formation of multiple creases observed in our experiments.

**Figure 10:**
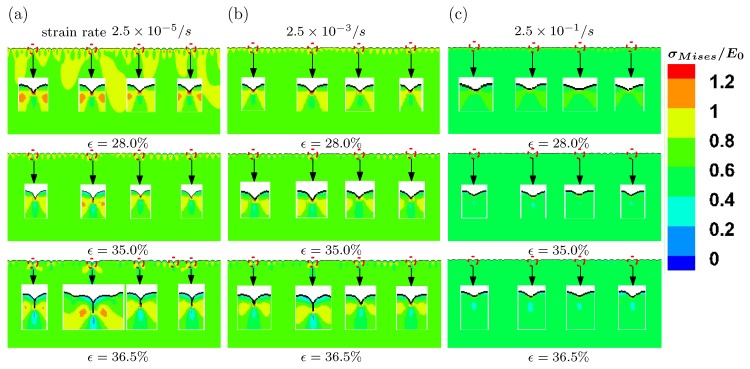
Evolution of surface morphology for a three-layer model at three levels of strains with different imposed strain rates. (**a**) $2.5\times 10^{-5}$/s; (**b**) $2.5\times 10^{-3}$/s; (**c**) $2.5\times 10^{-1}$/s.

We then examine the effect of viscosity on the formation of surface creases. We simulate the post-buckling behaviors under compression with three different levels of strain rates, namely $2.5\times 10^{-5}$, $2.5\times 10^{-3}$ and $2.5\times 10^{-1}$/s. The model parameters are the same as case III shown in Figure 9. The evolution of surface morphology under different levels of strains rates is presented in Figure 10. All the creasing patterns under different loading rates are very similar to each other. When the loading rate is lower, the cusps on surface are formed at strain 28%. However, with higher loading rate $2.5\times 10^{-1}$/s, the cusp is not evident at the same level of imposed strain. This indicates that the higher imposed loading rate can delay the occurrence of surface creases. Such an effect is more pronounced at compressive strain 36.5%. Under the lower strain rate $2.5\times 10^{-5}$/s, the length of self-contact region for the crease is larger, observed in the inserts of Figure 10, compared with that for higher strain rate $2.5\times 10^{-1}$/s.

**Figure 11:**
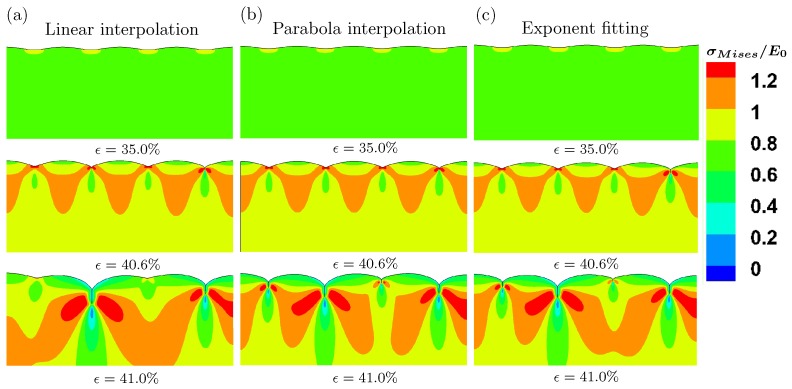
Evolution of surface morphology for multi-layer model at three levels of strains with different fitting methods for gradient modulus. (**a**) linear interpolation; (**b**) parabola interpolation; (**c**) exponential fitting.

According to a nanoindentation test, the modulus of silicon rubber varies along the height of specimen after cyclic pinching. Intuitively, the multi-layer model with gradient modulus is more appropriate to model the surface instability behaviors [36,37,38]. Three models with gradient modulus are shown in Figure 7c. We perform the buckling analysis first and then post-buckling analysis. The loading rate in terms of strain is $2.5\times 10^{-5}$/s. Figure 11 demonstrates the evolution of surface morphology under different levels of imposed strain for these three models with linear interpolation, parabola interpolation and exponential fitting of the experimentally measured moduli. When the imposed strain is 35%, only wrinkles can be observed on the surface. However, the amplitude of these wrinkles is larger than that of imperfections set on the surface before post-buckling analysis. The wrinkle forms persist until the imposed strain is around 40.6%. Then, the wrinkles evolve into creases suddenly at the strain 41%. Here, we can conclude that the gradient modulus can dramatically delay the forming of creases. Such a conclusion is consistent with Ref. [36]. Cao et al. showed that a gradient variation of the Young’s modulus generated by introducing nanoparticles along the depth direction can significantly increase the critical compressive strain in comparison with that without surface treatment and wrinkling may be prevented [36]. However, the results predicted by the model with gradient modulus are not comparable to our experiments, against our intuition. Although the gradient model appears to be more elaborate, it is difficult to describe and explain the multiple creases of silicone rubber under compression with the pre-imposed cyclic loading.

## 5. Conclusions

In this work, we have performed uniaxial compression tests on a cuboid silicone rubber with pre-imposed cyclic pinching. We find that multiple creases are formed on the surface, which are significantly different from what have been observed for the specimen without pre-imposed cyclic pinching. Nanoindentation measurements confirm that the modulus varies along the height of the specimen after pre-imposed cyclic pinching. With the measured moduli at different locations, we have built three-layer and multilayer FE models to explain the underlying physics involved during the formation of multiple creases observed in our experiments.

Our simulation results demonstrate that the formation of a thin layer on the top surface with a modulus a little larger than that at the bottom is an important factor for the observed multiple creases. The results also exhibit that if the modulus varies continuously along the height, it can delay the formation of surface creases. The same role is played by the viscosity of silicone rubber. However, the observations on the multilayer model are not comparable to our experiments. It appears that the modulus of silicon rubber varies in a piecewise way after the cyclic pinching because the three-layer model can explain the experimental results quite well.

In general, our results demonstrate a different surface instability behavior of silicone rubber with pre-imposed cyclic loading. A large amount of silicone rubber is now used in many engineering applications. They are usually operated under the cyclic loading conditions. The effect of these cyclic loadings or even fatigue on the surface instability should be given attention because the surface instability may greatly influence the reliability of these engineering structures with silicone rubber for long-term usage.
